# Etanercept Inhibits B Cell Differentiation by Regulating TNFRII/TRAF2/NF-*κ*B Signaling Pathway in Rheumatoid Arthritis

**DOI:** 10.3389/fphar.2020.00676

**Published:** 2020-05-12

**Authors:** Xiao-Yu Cai, Yue Zhu, Chen Wang, Xiao-Yu Tang, Le Han, Jin-Ling Shu, Xian-Zheng Zhang, Fa-Qin Liang, Jing-Ru Ge, Li Xu, Dan Mei, Ling-Ling Zhang, Wei Wei

**Affiliations:** Key Laboratory of Anti-inflammatory and Immune Medicine, Ministry of Education, Anhui Collaborative Innovation Center of Anti-Inflammatory and Immune Medicine, Institute of Clinical Pharmacology, Anhui Medical University, Hefei, China

**Keywords:** rheumatoid arthritis, etanercept, TNF-*α*, B cell differentiation, NF-*κ*B pathway

## Abstract

**Objective:**

To explore the role of B cells in rheumatoid arthritis (RA) and the potential effects and mechanisms of etanercept on B cells.

**Methods:**

In RA patients, the levels of tumor necrosis factor-α (TNF-α) and B cell activating factor (BAFF) were detected by ELISA. The percentage of B cell subsets was measured by flow cytometry. Laboratory indicators (rheumatoid factor, C-reactive protein, erythrocyte sedimentation rate) and clinical indicators (disease activity score in 28 joints, health assessment questionnaire score, swollen joint counts, tender joint counts) were measured. The correlation between B cell subsets and laboratory indicators or clinical indicators was analyzed. In mice, B cells proliferation was detected by CCK-8 kit. The expression of TNFRII and the percentage of B cell subsets in spleen were detected by flow cytometry. The expressions of TRAF2, p38, P-p38, p65, P-p65 in B cells were detected by WB.

**Results:**

The percentage of CD19^−^CD27^+^CD138^+^ plasma B cells was positively correlated with ESR or RF. Etanercept could decrease the percentage of CD19^+^ total B cells, CD19^+^CD27^+^ memory B cells and CD19^−^CD27^+^CD138^+^ plasma B cells, reduce the levels of TNF-α, BAFF, relieve clinical and laboratory indicators in RA patients. In addition, etanercept could inhibit the proliferation of B cells, bate the differentiation of transitional B cells to mature B cells, down-regulate the expression of TNFRII, TRAF2, P-p38, P-p65 in B cells.

**Conclusion:**

B cells act a key role in the pathogenesis of RA. Etanercept inhibits B cells differentiation by down-regulating TNFRII/TRAF2/NF-κB signaling pathway.

## Introduction

Rheumatoid arthritis (RA) is a chronic and systemic autoimmune disease characterized by synovitis, symmetry, and invasive arthritis of small joints such as wrist joint, metacarpophalangeal joint, proximal interphalangeal joint, and foot joint (Mcinnes and Schett, 2011). Loss of immune tolerance leads to the main cause of RA, and the excessive inflammation can lead to cartilage destruction and bone erosion, accompanied by systemic complications including cardiovascular disease (Magill et al., 2018). About 0.8–1% of adults were affected by RA, and some patients permanently lost their ability to work without effective treatment (Yao et al., 2018).

Complex interaction of immunocytes is involved in the pathogenesis of RA. B cells act a critical role in RA pathogenesis *via* producing rheumatoid factor (RF) and anticyclic-citrullinated peptide autoantibodies (anti-CCP) (Marston et al., 2010). In addition to antibody-dependent roles, B cells also produce cytokines that may enhance or weaken the function of other immunocytes. Most of all, as antigen presenting cells, B cells can recognize and present autoantigens to T cells (Zhang and Bridges, 2001). What’s more, the aggregation of B cells in synovium and cartilage is a histopathological feature of RA (Jimenez-Boj et al., 2005). B cells can form an ectopic lymph node structure and drive T cell activation and proliferation as part of synovial autoimmune response (Takemura et al., 2001a). Further, B cells produce receptor activator of NF-*κ*B ligand (RANKL) near the osteoclast precursors and promote bone destruction in a RANKL dependent way within synovium ectopic lymphoid structures (Sun et al., 2018). A previous study of our research group proved that abnormal activation of B cells participates in the pathological process of RA (Zhang et al., 2017). B cell activating factor (BAFF) belongs to the tumor necrosis factor superfamily, which is a crucial factor for B cell survival and maturation (Shin et al., 2018). Signaling of BAFF through BAFF receptor (BAFF-R) is involved in B cell maturation, activation, survival (Dorner et al., 2019). Above all, these studies indicated that B cells directly or indirectly participate in the progress of RA.

Tumor necrosis factor-α (TNF-α) is a major inducer of NF-*κ*B (Sayers et al., 2018). In different cell types, TNF-α can induce cell death or survival by activating NF-*κ*B (Barnhart and Peter, 2003; Micheau and Tschopp, 2003). TNF-α induces the expression of RANKL and directly promotes osteoclast differentiation synergizing with RANKL (Brennan and Mcinnes, 2008). Moreover, TNF-α induces inflammatory chemokine production, leading to immunocyte accumulation (Wei et al., 2005). In the past few decades, the use of biological drugs has greatly improved the condition of RA patients, especially those uncontrolled by conventional antirheumatic drugs (Scott, 2014). Etanercept, a tumor necrosis factor inhibitor, was the first approved antirheumatic biological agent and has made significant progress in the treatment of RA (Scott, 2014). The indications of etanercept include moderate and severe active RA, moderate and severe plaque psoriasis, and active ankylosing spondylitis (Emery et al., 2017).

The effect of etanercept on B cell function in RA patients is not clear. Does etanercept affect B cell subsets in RA patients, and how does etanercept affect the function of B cells? In order to study B cell subset changes in RA patients and to understand the potential role of etanercept on B cells, we analyzed the percentage of B cell subsets in the peripheral blood of RA patients with etanercept treatment. Our results show that etanercept could decrease the percentage of CD19^+^ total B cells, CD19^+^CD27^+^ memory B cells, and CD19^−^CD27^+^CD138^+^ plasma B cells. The purpose of this study is to elucidate the potential mechanism of etanercept in regulating B cell.

## Materials and Methods

### Drugs and Reagents

Etanercept (Shanghai Sunshine Guojian Pharmaceutical Co., Ltd., China); BAFF (R&D Systems, USA); TNF-α (novoprotein, China); fetal bovine serum (Zhejiang Tianhang Biotechnology, China). TRAF2(C-20), TRAF2-AF647, P-p38 (Santa Cruz Biotechnology, USA); p38 (Abcam, USA); p65 (Cell signaling Technology, USA); P-p65 (affinity Biosciences, USA); FITC Rat Anti-Mouse CD19, APC Mouse Anti-Human CD27, FITC Mouse Anti-Human CD138, PE Rat Anti-Mouse IgM, PE Mouse Anti-Human CD19 (BD Pharmingen, San Diego, CA, USA); TNFRI-APC, IgD-BV421 (BioLegend, San Diego, CA, USA); CD120b (TNF-RII)-APC (Miltenyi Biotec GmbH, Germany); Fetal bovine serum (Zhejiang Tianhang Biotechnology Co., Ltd, China); RPMI-1640 medium (HyClone, USA); Human BAFF ELISA kit, Human TNF-α ELISA kit (Nanjing Fcmacs Biotechnology Co., Ltd, China); Human lymphocyte separation solution, Mice lymphocyte separation solution (Shenzhen Dakco Biotechnology Co., Ltd, China); Cell Counting Kit (CCK-8/WST-8) Cell Proliferation and Activity Assay Kit: Biolite Biotech Trading Partners (Encinitas, CA, USA).

### Patient Selection and Sample Collection

Peripheral blood samples from RA patients were taken from the First Affiliated Hospital of Anhui Medical University. This study included 21 RA patients. In accordance with the American College of Rheumatology (ACR) classification criteria, patients with clinical symptoms lasting for at least 6 weeks and meeting four or more of the ACR classification criteria can be diagnosed as RA.

The ACR classification criteria were: (1) morning stiffness; (2) arthritis of at least three joint sites; (3) arthritis of the hand joints, at least one site of swelling; (4) symmetrical arthritis; (5) rheumatoid nodules; (6) RF abnormalities in serum; (7) radiological changes of typical RA in the joints of the anterior hand and wrist.

The selected RA patients were followed up (before treatment, after three months treatment, after six months treatment), and the following indexes were evaluated, including laboratory indicators: RF, C-reactive protein (CRP), erythrocyte sedimentation rate (ESR), and clinical indicators: disease activity score in 28 joints (DAS28), health assessment questionnaire score (HAQ), swollen joint counts (SJC), tender joint counts (TJC). Anticoagulant peripheral venous blood and procoagulant peripheral venous blood were collected before treatment, after three months treatment, and after six months treatment. Anticoagulant peripheral venous blood was used to separate peripheral blood mononuclear cells (PBMCs) for flow cytometry within 1 h. Serum was separated from procoagulant peripheral venous blood and stored at −80°C for subsequent experiments. Each participant provided informed consent before entering the study. This study was approved by the Anhui Medical University Biomedical Ethics Committee.

### Experimental Mice

Female DBA/1 mice were purchased from the Animal Experimental Center of Anhui Medical University. Animal protocols were approved by the Anhui Medical University Animal Ethics Committee.

### Detection of Laboratory Indicators

The automatic biochemical analyzer was used to measure the titer of serum RF and CRP. ESR value was detected by using Wei’s method. After blood collection, we gently mixed the blood in the blood sedimentation tube (300 × 2.5) upside down 8 to 10 times drew the above mixed blood to the “0” scale, stood the tube upright on the blood sediment rack, recorded at room temperature, observed the plasma height, and reported the ESR value in millimeters of the red blood cell sinking.

### Clinical Indexes of RA Patients

Clinical indexes of RA patients were collected: HAQ, SJC, TJC, DAS28. HAQ, SJC, and TJC were evaluated according to **Tables S1–S3**. DAS28 was evaluated according to the formula of DAS28 = 0.56× (TJC) + 0.28× (SJC) + 0.7× (ESR) + 0.014× (VAS), VAS = visual analogue scale.

### ACR Evaluation

ACR core criteria: TJC, SJC, VAS, Overall evaluation of disease activity (PtGA), Doctor’s activity on disease Overall Evaluation (PhGA), HAQ, Indicator for assessing disease activity: ESR or CRP. The 20% ACR standard (ACR20) refers to that the number of joint swelling and tenderness (28) was improved by 20% and at least three of the above parameters were improved by 20%. The 50% ACR standard (ACR50) refers to that the number of joint swelling and tenderness (28) was improved by 50% and at least three of the above parameters were improved by 50%.

### Detection of Human TNF-α and BAFF Levels by ELISA

The levels of human TNF-α and BAFF were measured by ELISA Kit according to the manufacturer’s instructions. Absorption was measured at 450 nm. Each sample was quantiﬁed with three replicates.

### Detection of Human B Cell Subsets by Flow Cytometry

Human peripheral blood mononuclear cells (HPBMCs) from RA patient were obtained by using human lymphocyte separation fluid and labeled with CD19-PE, CD27-APC, CD138-FITC incubated for 40 min at 4°C in dark. Then the cells were washed with PBS and analyzed by flow cytometry (FC500).

### Detection of Mouse Spleen B Cell Proliferation by CCK-8 Kit

Mice were sacrificed and the spleen was ground into single cell suspension in a super-clean table. Mice spleen mononuclear cells (MSMCs) were isolated by mouse lymphocyte isolation solution and added 100 μl to 96-well culture plate. Then MSMCs were stimulated by BAFF (20 ng/ml) and/or TNF-α (40 ng/ml) and incubated with etanercept (40 ng/ml or 80 ng/ml) for 48 h in 5% CO_2_ at 37°C. CCK-8 (10 µl) solution was added to a 96-well culture plate. The absorbance was read on the enzyme label within 1–4 h at 450 nm.

### Detection of Tumor Necrosis Factor Receptor II in Mouse B Cell Subsets by Flow Cytometry

Mice were sacrificed and the spleen was grinded into single cell suspension in a super-clean table. MSMCs were isolated by mouse lymphocyte isolation solution and incubated with surface antibody CD19-FITC, IgD-BV421, IgM-PE for 20 min at 4°C in dark. Subsequently, MSMCs were broken to incubate the intracellular antibody TNFRII-APC for 20 min in the dark and washed by PBS. Flow cytometry was used to detect the percentage of CD19^+^IgM^+^IgD^−^ type I transitional B cells, CD19^+^IgM^+^IgD^+^ type II transitional B cells, and CD19^+^IgD^+^IgM^−^ mature B cells.

### Detection of Mouse Mature B Cells by Flow Cytometry

Mice were sacrificed and the spleen was grinded into single cell suspension in a super-clean table. MSMCs were isolated by mouse lymphocyte isolation solution and added to a 96-well culture plate. Then MSMCs were stimulated by BAFF (20 ng/ml) and/or TNF-α (40 ng/ml) and incubated with etanercept (40 ng/ml or 80 ng/ml) for 48 h in 5% CO_2_ at 37°C. Subsequently, MSMCs were marked with CD19-FITC, IgD-PE, and IgM-APC antibody for 40 min at 4°C in the dark. The percentage of CD19^+^IgD^+^IgM^−^ mature B cells was detected by flow cytometry.

### Detection of TRAF2, p38, P-p38, p65, P-p65 Expression in Mouse Total B Cells by Western Blot

Mice were sacrificed and the spleen was grinded into single cell suspension in a super-clean table. MSMCs were isolated by mouse lymphocyte isolation solution and marked with CD19-FITC antibody for 40 min in the dark. Then CD19^+^ total B cells were isolated by flow sorting and were stimulated by BAFF or TNF-α in 24-well plates. Then CD19^+^ total B cells were incubated with etanercept for 48 h, lysed by RIPA lysis buffer (with PMSF 1% and phosphatase inhibitors 1%) at 4^°^C for 30 min. Proteins were centrifuged at 12,000r/min for 15 min at 4°C to remove residue, separated by 10% SDS-PAGE gel and transferred onto a PVDF Membrane. The membranes were blocked with 5% nonfat milk at 37°C for 2 h and then incubated with primary antibodies of rabbit monoclonal TRAF2 (1:1,000) or p38 (1:1,000) or P-p38 (1:500) or p65 (1:200) or P-p65 (1:200) and anti-*β*-actin (1:1,000) at 4^°^C for 12–16 h. Membranes were washed and incubated with secondary antibodies (1:10,000) at 37°C for 2 h. The protein bands were detected by chemiluminescence kit. Finally, the densities of the bands were quantified by ImageJ.

### Statistics

Data analysis was performed by SPSS16.0 software. The measured values of all experimental data were compared by mean and standard deviation (Mean ± Standard Error of Mean, X ± SD). One-way ANOVA was used to compare the significance of differences between groups. P < 0.05 meant statistical difference.

## Results

### Correlation Between Plasma B Cell Percentage and Laboratory Parameters Level in RA Patients

**Figure 1:**
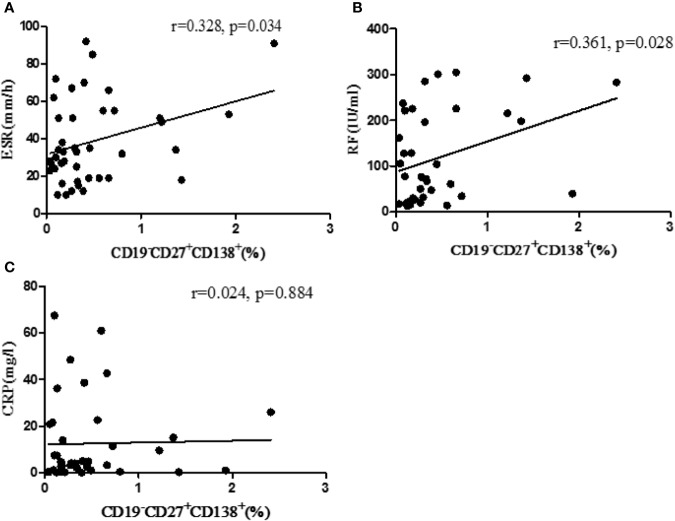
Correlation between the percentage of CD19^−^CD27^+^CD138^+^ B cells and laboratory parameters. **(A)** The correlation between percentage of B cells with ESR. **(B)** The correlation between the percentage of B cells with RF. **(C)** The correlation between the percentage of B cells with CRP. r, correlation coefficient; p, significant level. *p* < 0.05 indicates statistical significance.

RA patients were followed up (before treatment, after three months treatment, and after six months treatment). The levels of CD19^+^ total B cells, CD19^+^ CD27^+^ memory B cells, CD19^−^CD27^+^CD138^+^ plasma B cells, and laboratory indicators were detected. The results showed that the percentage of CD19^−^CD27^+^CD138^+^ plasma B cells was positively correlated with ESR (p = 0.034) and RF (p = 0.028) (**Figures 1A, B**). There was no correlation between the percentage of plasma B cells and CRP (*p* = 0.884) (**Figure 1C**).

### Etanercept Decreased B Cell Subsets Percentage in RA Patients

**Figure 2:**
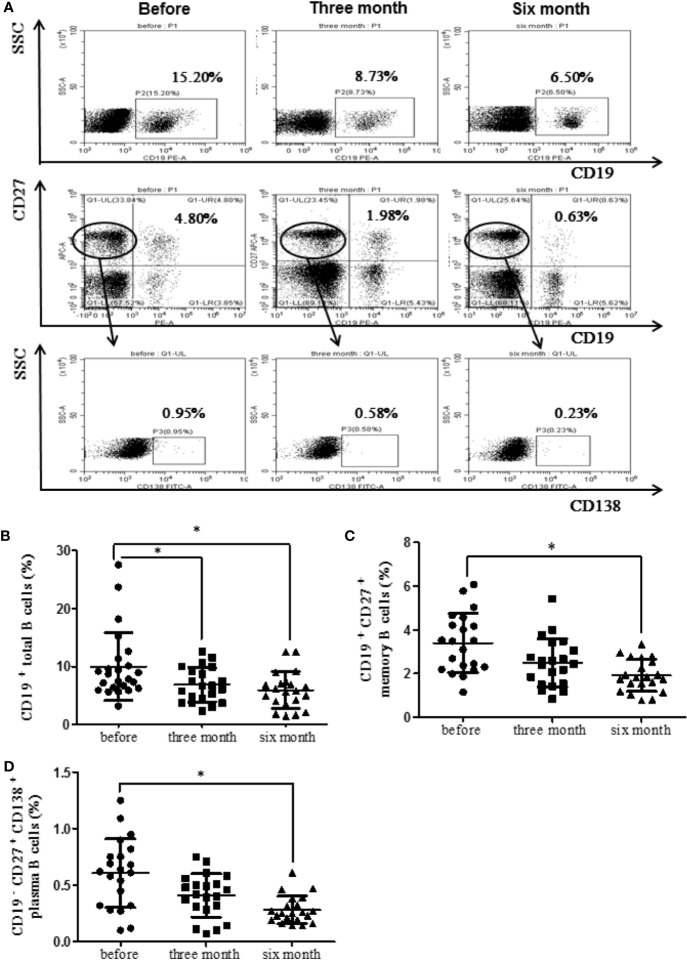
Etanercept could down-regulate the percentage of peripheral blood B cell subsets in patients with RA. **(A)** The representative flow cytometry graphs of one RA patients’ B cell subsets. **(B)** The percentage of CD19^+^ B cells was analyzed by flow cytometry after etanercept treatment. **(C)** The percentage of CD19^+^CD27^+^ B cells was analyzed by flow cytometry after etanercept treatment. **(D)** The percentage of CD19^−^CD27^+^CD138^+^ B cells was analyzed by flow cytometry after etanercept treatment. *p < 0.05.

RA patients were followed up (before treatment, after three months treatment and after six months treatment). The levels of CD19^+^ total B cells, CD19^+^CD27^+^ memory B cells, CD19^−^CD27^+^CD138^+^ plasma B cells were detected. The results showed that the percentage of CD19^+^ total B cells decreased significantly after three months and six months treatment with etanercept (*p* < 0.05) (**Figure 2B**). The percentage of CD19^+^CD27^+^ memory B cells and CD19^−^CD27^+^CD138^+^ plasma B cells decreased significantly after six months treatment with etanercept (*p* < 0.05) (**Figures 2C, D**). A flow chart of the peripheral blood B cell subsets in one of the RA patients is shown in **Figure 2A**.

### Etanercept Decreased TNF-α and BAFF Serum Levels in RA Patients

**Figure 3:**
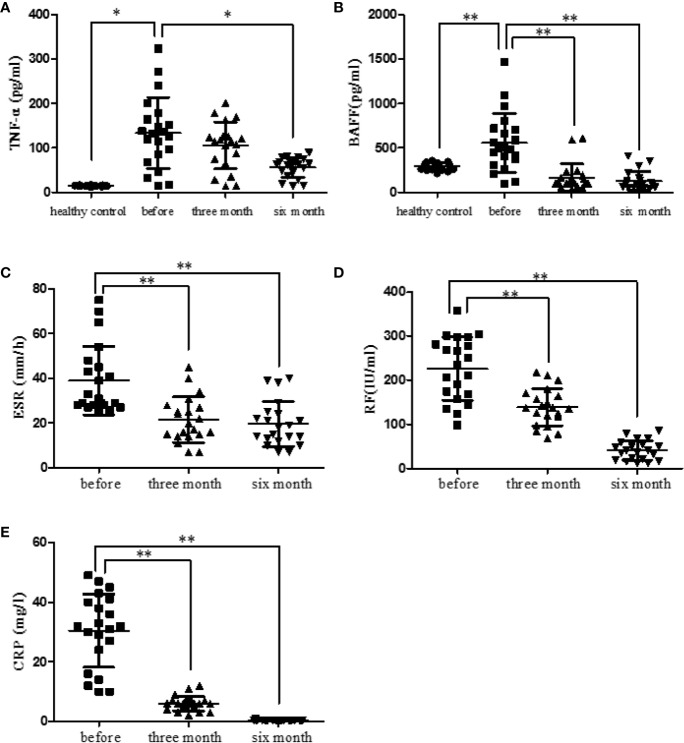
Etanercept down-regulates cytokine levels (TNF-α and BAFF) and laboratory indicators (ESR, CRP, and RF) in patients with RA. **(A)** The level of TNF-α in healthy people, RA patients, and RA patients treated with etanercept was detected by ELISA. **(B)** The level of BAFF in healthy people, RA patients, and RA patients treated with etanercept was detected by ELISA. **(C)** Change in ESR level after etanercept treatment. **(D)** Change in CRP level after etanercept treatment. **(E)** Change in RF level after etanercept treatment. *p < 0.05, **p < 0.01.

RA patients were followed up (before treatment, after three months treatment and after six months treatment). The levels of serum TNF-α and BAFF in healthy and RA patients were measured using ELISA kits. Results displayed that TNF-α level was significantly elevated in RA patients (*p* < 0.05). After treatment with etanercept, the level of TNF-α decreased, especially after six months of the treatment (*p* < 0.05) (**Figure 3A**). BAFF level was also elevated in RA patients compared with that in healthy people (*p* < 0.01). After treatment with etanercept, BAFF level decreased, especially after three months and six months of the treatment (*p* < 0.01) (**Figure 3B**).

### Etanercept Decreased Laboratory Parameters in RA Patients

RA patients were followed up (before treatment, after three months treatment and after six months treatment). Laboratory parameters of RA patients were examined and statistically analyzed. Etanercept could significantly decrease the levels of ESR (*p* < 0.01), CRP (*p* < 0.01), and RF (*p* < 0.01) after three months and six months of the treatment (**Figures 3C–E**).

### Etanercept Decreased Clinical Indexes in RA Patients

**Figure 4:**
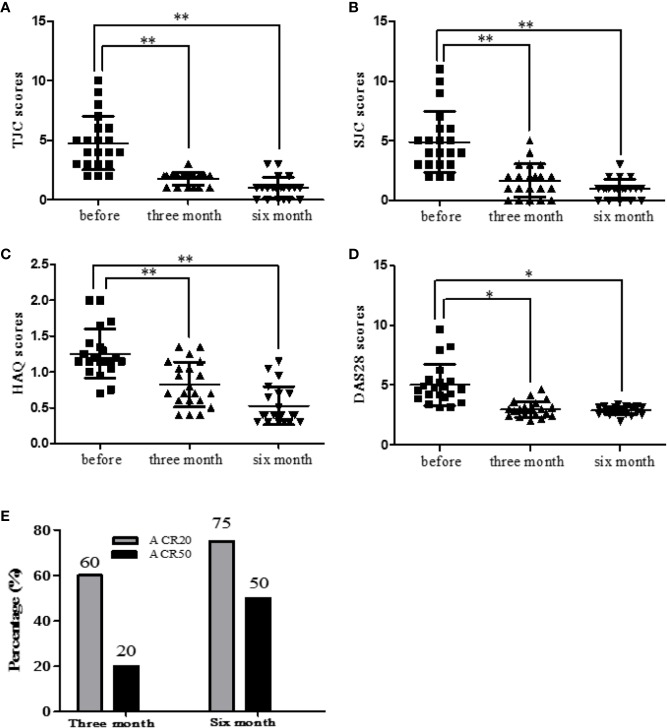
Etanercept down-regulates clinical indicators (HAQ, SJC, TJC, and DAS28) in patients with RA. **(A)** Change in the level of TJC scores after etanercept treatment. **(B)** Change in the level of SJC scores after etanercept treatment. **(C)** Change in the level of HAQ scores after etanercept treatment. **(D)** Change in the level of DAS28 scores after etanercept treatment. **(E)** Treatment with etanercept, the percentage of RA patients who have improved to ACR20 and ACR50 standards. *p < 0.05, **p < 0.01.

RA patients were followed up (before treatment, after three months treatment and after six months treatment). The clinical indexes of RA patients were collected and statistically analyzed. Etanercept could significantly decrease the levels of HAQ (*p* < 0.01), SJC (*p* < 0.01), TJC (*p* < 0.01), and DAS28 (*p* < 0.05) after three months and six months of the treatment (**Figures 4A–D**).

### Percentage of RA Patients Reaching ACR20/50 After Etanercept Treatment

After three months of etanercept treatment, 60% patients’ condition improved to ACR20 standard, and 20% patients’ condition improved to ACR50 standard. Six months after etanercept treatment, 75% of patients achieved ACR20, and 50% of patients achieved ACR50 (**Figure 4E**).

### Etanercept Inhibited TNF-α-Stimulated Spleen B Cell Proliferation in Mice

**Figure 5:**
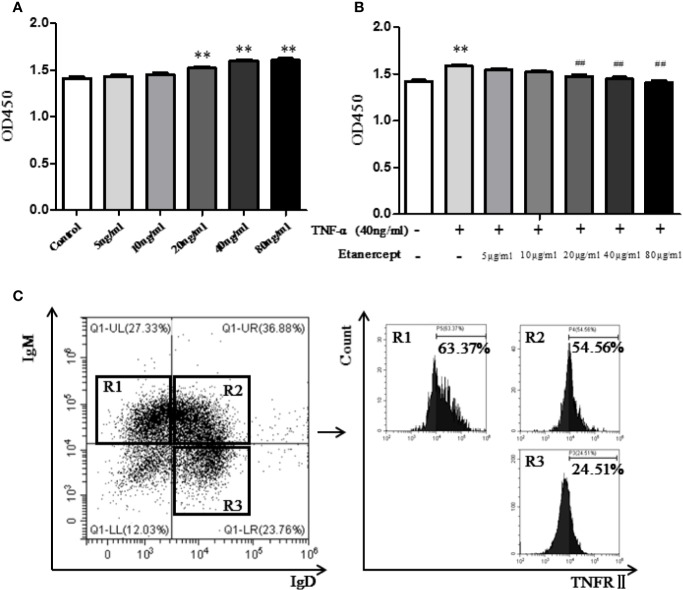
**(A)** The effect of different concentrations of TNF-α on the proliferation of B cells was detected by CCK-8. **(B)** The effect of different concentrations of etanercept on the proliferation of B cells stimulated by TNF-α was detected by CCK-8. **(C)** Expression of TNFRII on type I transitional B cells (CD19^+^IgM^+^IgD^−^), type II transitional B cells (CD19^+^IgM^+^IgD^+^), and mature B cells (CD19^+^IgD^+^IgM^−^) by flow cytometry. ^##^*p* < 0.01 *vs* TNF-α group, **p < 0.01 *vs* Control group.

The CCK-8 method was used to detect B cell proliferation. The results displayed that TNF-α promoted B cell proliferation at the concentration of 20 ng/ml and 40 ng/ml, especially 40 ng/ml (**Figure 5A**). Etanercept (> 20 µg/ml) inhibited spleen B cell proliferation in TNF-α-stimulated mice compared with the TNF-α-stimulated group, especially 40 µg/ml (**Figure 5B**).

### TNFR II Expression in Mouse B Cell Subsets

Flow cytometry was used to analyze the expression of TNFRII on mouse spleen B cells. The results showed that TNFRII was expressed on CD19^+^IgM^+^IgD^−^ type I transitional B cells, CD19^+^IgM^+^IgD^+^ type II transitional B cells, CD19^+^IgD^+^IgM^−^ mature B cells (**Figure 5C**). In addition, the expression levels of TNFRII on CD19^+^IgM^+^IgD^−^ type I transitional B cells and CD19^+^IgM^+^IgD^+^ type II transitional B cells were higher than that on CD19^+^IgD^+^IgM^−^ mature B cells.

### Etanercept Inhibited TNFR II Expression on Type II Transitional B Cells in Mice

**Figure 6:**
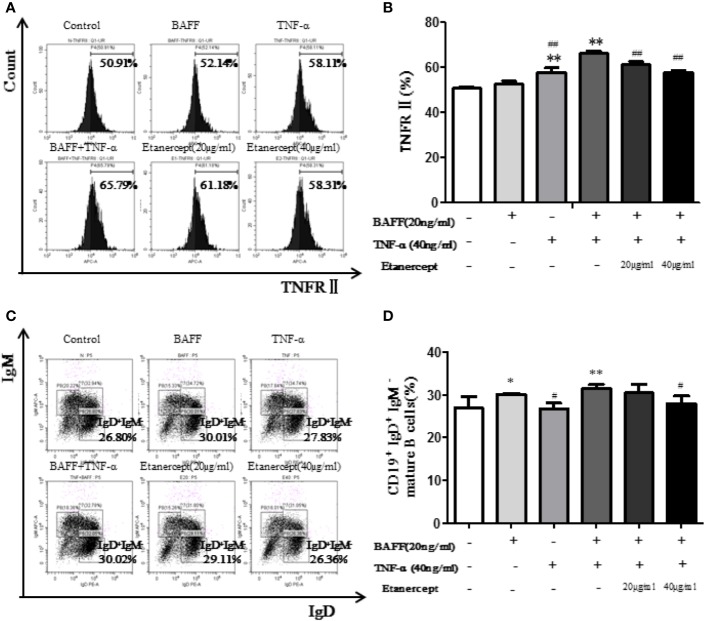
Etanercept inhibits the transition of transitional B cells to mature B cells. **(A)** The representative flow cytometry graphs of TNFRII expression on type II transitional B cells in different groups. **(B)** The expression of TNFRII on type II transitional B cells IS compared between different groups. **(C)** The representative flow cytometry graphs of mature B cells’ percentage in different groups. **(D)** Percentage of mature B cells was compared between different groups. *p < 0.05 *vs* Control group, **p < 0.01 *vs* Control group, ^#^*p* < 0.05 *vs* BAFF+TNF-α group, ^##^*p* < 0.01 *vs* BAFF+TNF-α group.

The expression of TNFRII on CD19^+^IgM^+^IgD^+^ type II transitional B cells was analyzed by flow cytometry. The results displayed that TNFRII percentage in the TNF-α + BAFF group was higher than that in the blank control group. Etanercept (20 µg/ml and 40 µg/ml) could significantly down-regulate the expression of TNFRII (*p* < 0.01) (**Figures 6A, B**).

### Etanercept Inhibited the Transition of Type II Transitional B Cells to Mature B Cells in Mice

The percentage of B cell subsets was analyzed by flow cytometry. The results displayed the CD19^+^IgD^+^IgM^−^ mature B cell percentage in the TNF-α + BAFF group was higher than that in the blank control group (*p* < 0.01). Etanercept at a concentration of 40 μg/ml could significantly decrease the percentage of CD19^+^IgD^+^IgM^−^ mature B cells (*p* < 0.05) (**Figures 6C, D**).

### Etanercept Inhibited TRAF2, P-p38, and P-p65 Expression in BAFF and TNF-α Stimulated Mouse Total B Cells

**Figure 7:**
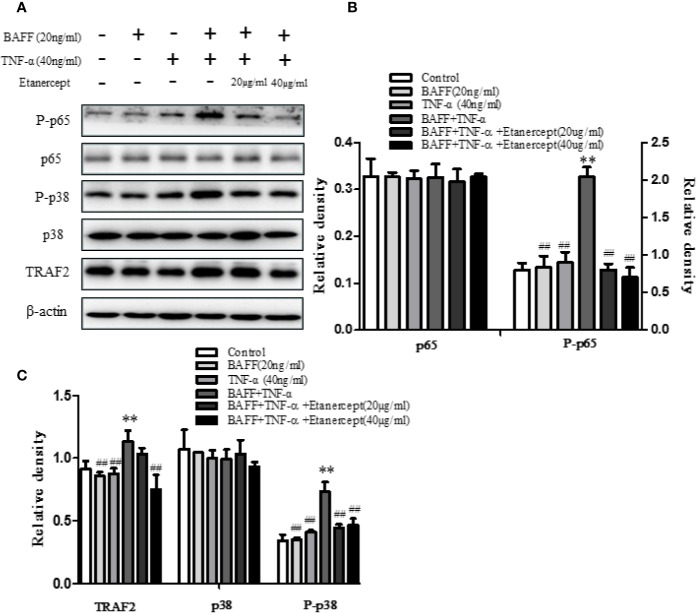
The effect of etanercept on the expressions of TRAF2, p38, P-p38, p65, P-p65 in B cells stimulated by BAFF and TNF-α. **(A)** The representative graphs of the expressions of TRAF2, p38, P-p38, p65, P-p65 in different groups. **(B)** The cartogram of the expressions of p65 and P-p65 in different groups. **(C)** The cartogram of the expressions of TRAF2, p38, and P-p38 in different groups. **p < 0.01 *vs* Control group. ^##^*p* < 0.05 *vs* BAFF + TNF-α group.

The expressions of TRAF2, p38, P-p38, p65, P-p65 in CD19^+^ total B cells were analyzed. The results displayed that costimulation of BAFF and TNF-α boosted the expressions of TRAF2, P-p38, and P-p65 but had no effect on the expressions of p38 and p65. Etanercept (20 µg/ml) could reduce the high expressions of P-p38, P-p65 in B cells stimulated by BAFF and TNF-α (*p* < 0.01). Etanercept (40 µg/ml) could significantly reduce the high expressions of TRAF2, P-p38, P-p65 in B cells stimulated by BAFF and TNF-α. Etanercept had no effect on the expressions of p38 and p65 (*p* > 0.05) (**Figures 7A–C**).

## Discussion

Genetic factors and environmental factors have been attributed to RA, but the triggers that initiate the disease are unknown (Whang and Chang, 2014). In recent years, more and more attention has been paid to the role of B cells in the immunopathogenesis of RA. B cells provide the necessary costimulatory signals for clonal expansion and effector functions of CD4^+^ T cells (Shaw et al., 2003). T cell activation is critically dependent on the presence of B cells, which is an important component of RA pathogenesis (Takemura et al., 2001b). B cells play a critical role in RA *via* RF and anti-CCP production. The occurrence of RF may precede the occurrence of RA clinical symptoms (Corsiero et al., 2014). RF, known as IgG antibody specific for IgG Fc, can provide positive feedback signals to pre-B cells *via* self-polymerization acquiring complement component 3d (C3d) (Edwards and Cambridge, 2006). ESR and CRP are two commonly used indicators for the identification and evaluation of RA (Walsh et al., 1979). RA patients with abnormal ESR level above 100 mm/h usually have a poor prognosis (Brigden, 1999). ESR is mainly used in the clinical observation of the RA patient’s condition change. CRP, discovered in 1930, is a protein that binds to C-polysaccharide of pneumococcus. CRP level is usually very low in healthy people (< 5 mg/L), but it can reach its peak within 48 h of inflammation. During inflammation, the level of CRP can be increased by tens or even hundreds of times (Laiho et al., 2001). In this study, we discovered that CD19^−^CD27^+^CD138^+^ plasma B cell level was positively correlated with RF and ESR titers, indicating that CD19^−^CD27^+^CD138^+^ plasma B cell subsets may play an important role in RA.

Etanercept can significantly reduce the clinical symptoms of RA patients and improve their quality of life. Long-term use of etanercept in RA patients is safe and effective in clinic (Emery et al., 2008). TNF-α is widely present in the serum and arthritis synovium of RA patients, acting on a variety of cells in joint space, amplifying and cascading inflammatory process (Moelants et al., 2013). TNF-α induces activation and recruitment of immunocytes in Synovia of RA patients (Romas et al., 2002). Also, TNF-α induces RANKL expression and promotes osteoclast differentiation and bone loss in a synergistic manner with RANKL. BAFF is a survival factor of B cells and a member of the tumor necrosis factor superfamily, which plays a crucial role in regulating the homeostasis and maturation of B cells (Loder et al., 1999; Wei et al., 2015). In this experiment, the levels of serum TNF-α and BAFF in RA patients and in healthy people were measured. The result showed that the levels of TNF-α and BAFF were significantly higher than that in healthy people; etanercept could significantly reduce the levels of TNF-α and BAFF. Above all, the results suggest that TNF may play a non-ignorable role in the pathological process of RA.

The increase in the number of circulating B cells may be owing to the continuous activation of B cells (Lindenau et al., 2003). Previous studies by our team have found that abnormal activation of B cells does exist in RA patients (Zhang et al., 2017). This study showed that the percentage of CD19^+^ total B cells, CD19^+^CD27^+^ memory B cells, and CD19^−^CD27^+^CD138^+^ plasma B cells, the levels of TNF-α, BAFF, laboratory parameters, and clinical indicators of RA patients all decreased after etanercept treatment. The above results suggest that etanercept may affect the abnormal immune response of RA by inhibiting the abnormal activation of B cells, thereby improving the clinical symptoms of RA patients and restoring abnormal laboratory indicators.

The development of B cells is an orderly process, which can be divided into five stages: pro-B cells, pre-B cells, immature B cells, transitional B cells, and CD19^+^IgD^+^IgM^−^ mature B cells. The first three stages are in bone marrow, and the last two stages are mainly in the spleen. Spleen mature B cells eventually differentiate into peripheral plasma B cells and memory B cells to exert immune function (Niiro and Clark, 2002). Transitional B cells mainly include two subgroups: CD19^+^IgM^+^IgD^−^ type I transitional B cells and CD19^+^IgM^+^IgD^+^ type II transitional B cells. Peripheral blood B cells are mainly derived from CD19^+^IgM^+^IgD^+^ type II transitional B cells (Loder et al., 1999). A previous study in our laboratory has shown that etanercept could reduce the percentage of CD19^+^ total B cells, CD19^+^CD27^+^ memory B cells, and CD19^−^CD27^+^CD138^+^ plasma B cells in CIA mice (Shu et al., 2019). In order to investigate whether etanercept has an effect on the differentiation of B cells, we further designed a series of animal experiments. First, we detected the expression of TNFR in B cells by flow cytometry. The result showed that TNFRII was mainly expressed on CD19^+^IgM^+^IgD^−^ type I transitional B cells, CD19^+^IgM^+^IgD^+^ type II transitional B cells and CD19^+^IgD^+^IgM^−^ mature B cells. TNFRII levels on CD19^+^IgM^+^IgD^−^ type I transitional B cells and CD19^+^IgM^+^IgD^+^ type II transitional B cells were higher than that on CD19^+^IgD^+^IgM^−^ mature B cells. Therefore, we hypothesized that etanercept may inhibit the differentiation of CD19^+^IgM^+^IgD^+^ type II transitional into CD19^+^IgD^+^IgM^−^ mature B cells through TNFRII. Subsequently, MSMCs were stimulated by BAFF (20 ng/ml) and/or TNF-α (40 ng/ml) and treated by etanercept. Flow cytometry result showed that etanercept could inhibit the high expression level of TNFRII on CD19^+^IgM^+^IgD^+^ type II transitional B cells stimulated by TNF-α and BAFF. In addition, the percentage of CD19^+^IgD^+^IgM^−^ mature B cells decreased after etanercept treatment. TNF-α is the main inducing factor of NF-κB. TNF-α can induce cell death or survival through activation of NF-κB in different cell types. In this study, WB results showed that etanercept could significantly reduce the high expressions of TRAF2, P-p38, P-p65 in CD19^+^ total B cells. Therefore, we hypothesized that etanercept could inhibit B cell activation and differentiation by acting on TNFRII/TRAF2/NF-κB signaling pathway in transitional B cells.

## Conclusion

In summary, B cells play a key role in the pathogenesis of RA. Abnormal activation and differentiation of B cells are involved in the pathological process of RA. As a kind of targeted biologic agent with good clinical effect, etanercept can affect the abnormal immune response of RA by down-regulating abnormally elevated inflammatory cytokines and activated B cell subsets, thereby improving clinical symptoms and recovery of abnormal laboratory indicators. Etanercept could inhibit B cell differentiation by down-regulating the TNFRII/TRAF2/NF-κB signal pathway. This study provides an important experimental basis for revealing the pathogenesis of B cells in RA. Further, this study clarifies the potential mechanism of etanercept in the treatment of RA.

## Data Availability Statement

All datasets generated for this study are included in the article/**Supplementary Material**.

## Ethics Statement

The studies involving human participants were reviewed and approved by Anhui Medical University Biomedical Ethics Committee. The patients/participants provided their written informed consent to participate in this study. The animal study was reviewed and approved by Animal Ethics Committee of Anhui Medical University. Written informed consent was obtained from the individual(s) for the publication of any potentially identifiable images or data included in this article.

## Author Contributions

X-YC analyzed the data and wrote this manuscript. X-YC, YZ, and CW performed the experiments and helped to revise this manuscript. X-YT , LH, J-LS, XZZ, and F-QL aided in processing the data. LX, J-RG, and DM aided in collection of materials. L-LZ and WW, corresponding the authors, conceived and designed the experiments.

## Funding

This study was supported by the National Natural Science Foundation of China (grant number U1803129, 81803538 and 81973332).

## Conflict of Interest

The authors declare that the research was conducted in the absence of any commercial or financial relationships that could be construed as a potential conflict of interest.
