# Supplementary material for: Etanercept Inhibits B Cell Differentiation by Regulating TNFRII/TRAF2/NF-κB Signaling Pathway in Rheumatoid Arthritis
Source: Front Pharmacol. 2020 May 12;11:676. doi: 10.3389/fphar.2020.00676 (PMC7235293; doi:10.3389/fphar.2020.00676)
Supplement: Supplementary file 1 [file DataSheet_1.pdf]

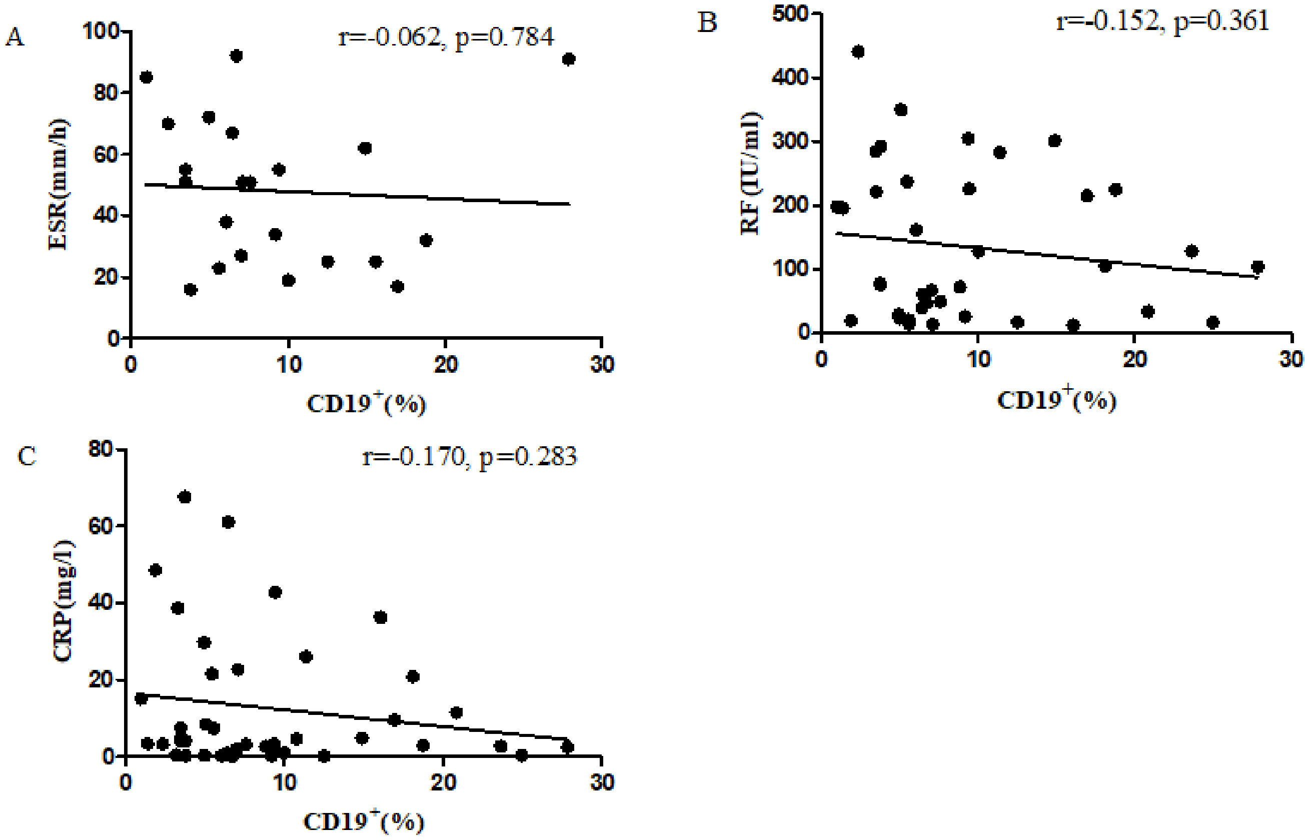

Supplementary Figure 1. Correlation between the percentage of $CD19^+$ B cells and laboratory parameters. (A) The correlation between percentage of $CD19^+$ B cells with ESR. (B) The correlation between percentage of $CD19^+$ B cells with RF. (C) The correlation between percentage of $CD19^+$ B cells with CRP. r, correlation coefficient; p, significant level. $p < 0.05$ indicates statistical significance.

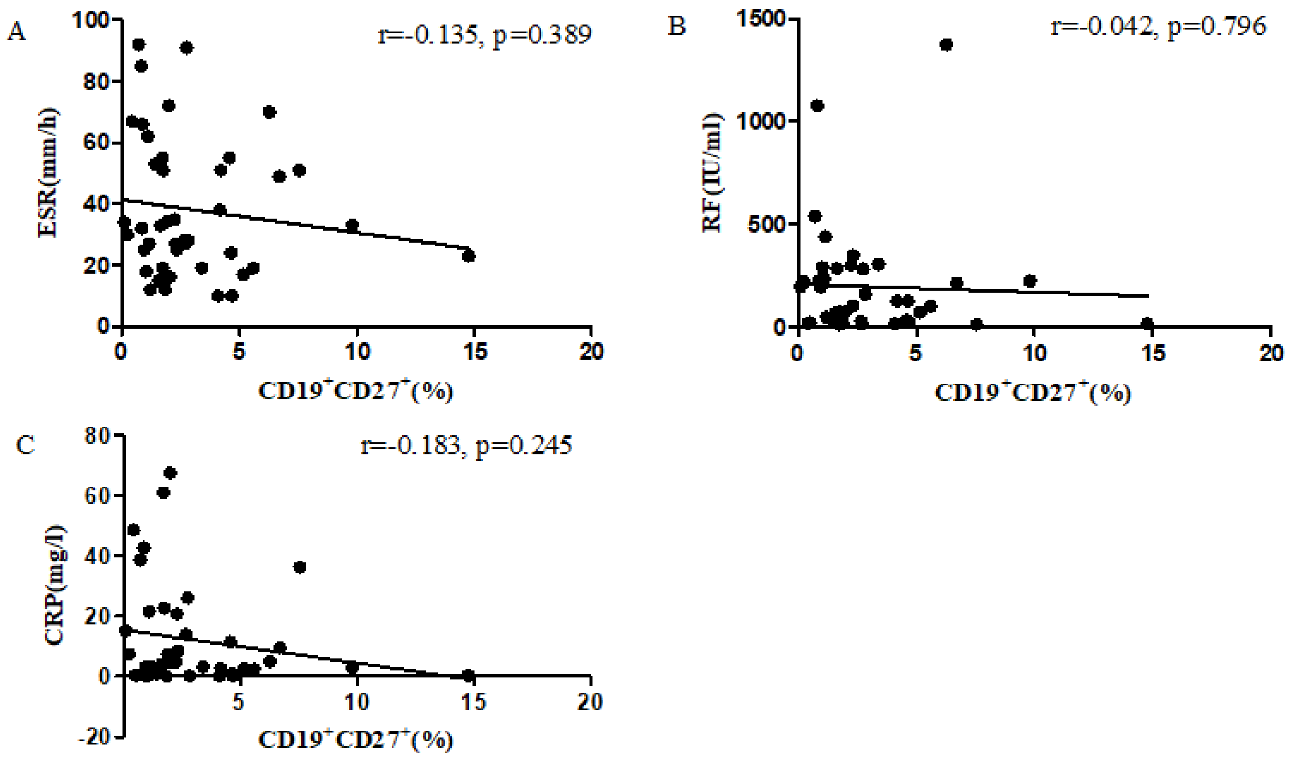

Supplementary Figure 2. Correlation between the percentage of $CD19^{+}CD27^{+}$ B cells and laboratory parameters. (A) The correlation between percentage of $CD19^{+}CD27^{+}$ B cells with ESR. (B) The correlation between percentage of $CD19^{+}CD27^{+}$ B cells with RF. (C) The correlation between percentage of $CD19^{+}CD27^{+}$ B cells with CRP. r, correlation coefficient; p, significant level. $p < 0.05$ indicates statistical significance.
