# Supplementary material for: Etanercept Inhibits B Cell Differentiation by Regulating TNFRII/TRAF2/NF-κB Signaling Pathway in Rheumatoid Arthritis
Source: Front Pharmacol. 2020 May 12;11:676. doi: 10.3389/fphar.2020.00676 (PMC7235293; doi:10.3389/fphar.2020.00676)
Supplement: Supplementary file 2 [file DataSheet_2.pdf]

Supplementary table 1: Health assessment questionnaire

RA patient HAQ total scores:_______ average scores: _______

During the past week, you performed the following activities: 0 = no difficulty; 1 = somewhat difficult; 2 = very difficult; 3 = impossible.					
1	Dressing (tie shoelaces and buttons)		11	Get up or sit down from the toilet	
2	Comb one's hair		12	Bent up and pick up things on the ground	
3	Stand up from the chair without hand support		13	Reach out and remove the coat hanger	
4	Go to bed, get up		14	Switch faucet	
5	Raise a glass of drinking water		15	Getting on and off the car	
6	Cut vegetables		16	Go shopping	
7	Unscrew the bottle cap		17	Doing housework such as cleaning	
8	Walking flat outdoors		18	Walk one kilometer	
9	Upper five steps		19	Participate in favorite activities	
10	Bathe and dry the body		20	Sleep well at night	

Supplementary table 2: Patient's overall pain visual analogue scale

Patient's overall pain (VAS): _____ cm (0 = no pain, 10 = pain is unbearable)

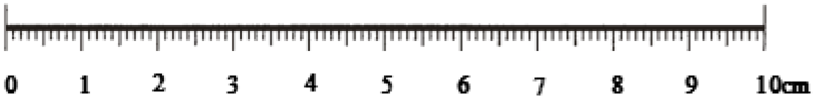

Among them, 0 points: no pain; 3 points or less: slight pain, can bear; 4 points - 6 points: the patient's pain and affect sleep, still can bear; 7 points -10 points: the patient has gradually strong pain, pain Unbearable, affecting appetite and affecting sleep.

Supplementary table 3: Tender joint counts and swollen joint counts questionnaire

Left side			Joint Name	Right side		
Tenderness<br>Y N N/A	Swelling<br>Y N N/A	NE		NE	Tenderness<br>Y N N/A	Swelling<br>Y N N/A
□ □ □	□ □ □	□	*Shoulder joint*	□	□ □ □	□ □ □
□ □ □	□ □ □	□	*Elbow joint*	□	□ □ □	□ □ □
□ □ □	□ □ □	□	*wrist joints*	□	□ □ □	□ □ □
□ □ □	□ □ □	□	*First metacarpophalangeal joint*	□	□ □ □	□ □ □
□ □ □	□ □ □	□	*Second metacarpophalangeal joint*	□	□ □ □	□ □ □
□ □ □	□ □ □	□	*Third metacarpophalangeal joint*	□	□ □ □	□ □ □
□ □ □	□ □ □	□	*Fourth metacarpo phalangeal joint*	□	□ □ □	□ □ □
□ □ □	□ □ □	□	*Fifth metacarpophalangeal joint*	□	□ □ □	□ □ □
□ □ □	□ □ □	□	*Interphalangeal joint*	□	□ □ □	□ □ □
□ □ □	□ □ □	□	*Second proximal knuckle*	□	□ □ □	□ □ □
□ □ □	□ □ □	□	*Third proximal knuckle*	□	□ □ □	□ □ □
□ □ □	□ □ □	□	*Fourth proximal knuckle*	□	□ □ □	□ □ □
□ □ □	□ □ □	□	*Fifth proximal knuckle*	□	□ □ □	□ □ □
□ □ □	□ □ □	□	*Knee joint*	□	□ □ □	□ □ □
